# Impact of ionic imbalances on rat cardiomyocyte function in pulmonary arterial hypertension: insights from energy dispersive X-ray spectroscopy and scanning electron microscopy analysis

**DOI:** 10.1007/s00421-026-06175-z

**Published:** 2026-02-28

**Authors:** Leôncio Lopes Soares, Luciano Bernardes Leite, Bruno Rocha Avila Pelozin, Sebastião Felipe Ferreira Costa, Luiz Otávio Guimarães-Ervilha, Renê Chagas da Silva, Mariana Machado-Neves, Emily Correna Carlo Reis, Tiago Fernandes, Edilamar Menezes Oliveira, Antônio José Natali

**Affiliations:** 1 Department of Physical Education, Federal University of Juiz de Fora, Governador Valadares, MG Brazil; 2 Department of Physical Education, Federal University of Viçosa, Viçosa, MG Brazil; 3 School of Physical Education and Sports, University of São Paulo, São Paulo, SP Brazil; 4 Department of General Biology, Federal University of Viçosa, Viçosa, MG Brazil; 5 Department of Physics, Federal University of Viçosa, Viçosa, MG Brazil; 6 Department of Veterinary Medicine, Federal University of Viçosa, Viçosa, MG Brazil

**Keywords:** Pulmonary hypertension, Pathological remodeling, Heart failure, Right ventricle, Cardiomyocytes

## Abstract

Calcium, sodium and potassium are essential for the contractile function of cardiomyocytes. In pulmonary arterial hypertension (PAH), intracellular calcium dynamics are impaired, affecting contractility and leading to heart failure. Energy-dispersive X-ray spectroscopy associated with scanning electron microscopy (EDS-SEM) technique can be useful for mapping the distribution of these minerals in cardiac tissue. This study aimed to investigate chronic ionic imbalances in the cardiac tissue of a monocrotaline (MCT) induced PAH model, using EDS-SEM, and to evaluate the impact of these imbalances on the contractile function of isolated RV myocytes. Twenty-eight Wistar rats were assigned to Control and PAH groups, with PAH induced by MCT. Right ventricular (RV) function was assessed by echocardiography at day 24. On day 25, cardiac tissue was analyzed by EDS-SEM to quantify electrolytes and by Western blotting, and RV myocyte contractility was measured. Student’s t-test was performed to compare the groups. Animals with PAH showed reduced final weight, increased cardiac and RV weight, and reduced RV systolic function compared to controls. EDS-SEM analysis revealed lower Ca and Na density in cardiac tissue of PAH animals. The expression of Ca^2+^ regulatory proteins was reduced in the PAH group, and the contractility of isolated myocytes was impaired, exhibiting decreased amplitude, contraction velocity, and relaxation velocity. In conclusion, the study demonstrates that MCT-induced ionic imbalances, specifically reduced Ca and Na, disrupt excitation–contraction coupling proteins, leading to impaired cardiomyocyte contractility and contributing to the RV dysfunction observed in PAH.

## Introduction

Ionic homeostasis, particularly calcium, sodium, and potassium, are crucial for the optimal contractile function of cardiomyocytes and the entire heart (Nattel and Li 2000; Priest and McDermott 2015). Alterations in the levels of these minerals can occur in cardiomyopathies, subsequently compromising the contractility of cardiomyocytes (Bers 2002). For example, in pulmonary arterial hypertension (PAH), a rare disease with an unfavorable prognosis that often results in the patient mortality within a short timeframe, there are notable impairments in intracellular Ca^2^⁺ dynamics in the right ventricle (RV) (Soares et al. 2023, 2019). Reduction in intracellular Ca^2+^ may be associated with dysfunctions of excitation–contraction coupling proteins [i.e. type 2 ryanodine receptors (RyR2), Phospholamban (PLB), sarcoplasmic reticulum (SR) Ca^2+^-ATPase (SERCA2) and sodium/calcium exchanger (NCX1)] (Bers 2002) and that in PAH can lead to impairment in the contractility of RV cardiomyocytes, resulting in heart failure, which is the leading cause of death among these patients (Soares et al. 2023; Arrigo et al. 2024). However, while impairments in intracellular Ca^2+^ handling mechanisms are well-established, the overall elemental composition and distribution of ions within the cardiac tissue matrix remain unexplored. Specifically, it is unclear whether the structural remodeling of the RV is accompanied by quantitative shifts in tissue-level ionic reserves, which could be accurately assessed using microanalytical techniques.

Energy dispersive X-ray spectroscopy (EDS) associated with scanning electron microscopes (SEM) offers valuable potential for spatial mapping and evaluating the relative distribution of chemical elements in biological tissues, including cardiac tissue (Novaes et al. 2013; Patri et al. 2009). In this method, the sample is bombarded with a high-voltage electron beam. An interaction between the sample and the electron beam causes emission of radiation in the X-ray range, which is characteristic of an element. This enables rapid qualitative and quantitative analysis based on the intensity of the energy emitted (Khan et al. 2020). In the context of PAH, applying EDS-SEM provides a distinct advantage over traditional bulk assays by allowing the assessment of the in situ ionic landscape. This approach is particularly effective for evaluating changes in calcium (Ca), sodium (Na) and potassium (K) levels within the cardiac tissue architecture, providing crucial data on how mineral imbalances may correlate with the structural and functional remodeling of the organ.

Importantly, the significance of these ionic imbalances is not limited to experimental models but is central to the pathophysiology of human heart failure. Previous studies have established the critical role of Na + , K + -pump regulation and precise ionic homeostasis in determining myocardial contractility and fatigue resistance (Sejersted and Sjøgaard 2000; Schwinger et al. 2003; Swift et al. 2007; Despa and Bers 2013). Dysregulation of these mechanisms is a hallmark of heart failure. Therefore, validating microanalytical techniques like EDS-SEM in animal models holds significant translational potential, paving the way for the detailed elemental analysis of human endomyocardial biopsies to detect early ionic remodeling in PAH.

Therefore, the aim of this study was to investigate ionic imbalances in the cardiac tissue of a monocrotaline (MCT)-induced PAH model, using EDS-SEM, and to evaluate the impact of these imbalances on the contractile function of isolated RV myocytes.

## Methods

### Study design

Twenty-eight male Wistar rats, aged 2 months and weighing ~ 200 g, were randomly assigned to two experimental groups: Control (n = 14) and PAH (n = 14). All animals were housed in a temperature-controlled room (approximately 22 °C) with 60% relative humidity, maintained on a 12/12-h light/dark cycle, and provided with free access to water and standard rodent chow. The experimental protocol was approved by the Ethics Committee on Animal Use of the Federal University of Viçosa (CEUA/UFV; approval number 11/2022), in accordance with the Guide for the Care and Use of Laboratory Animals.

### Induction of experimental pulmonary arterial hypertension

For the induction of experimental PAH, animals in the PAH group received a single intraperitoneal injection of 60 mg/kg body weight of MCT (Sigma-Aldrich, St. Louis, MO, USA) dissolved in saline (140 mM NaCl; pH 7.4). Control animals received an equivalent volume of saline (140 mM NaCl; pH 7.4) (Natali et al. 2015).

### Echocardiography

The echocardiographic evaluation was performed on the 24th day after the administration of MCT. The animals were anesthetized with 1.5% isoflurane dissolved in 100% oxygen at a constant flow of 1 L/min (Isoflurane, BioChimico, RJ, Brazil). The images were acquired with the animals in the lateral decubitus position. Two-dimensional studies with a fast-sampling rate of 120 fps in M mode were performed using the MyLabTM30 ultrasound system (Esaote, Genoa, Italia) and 11 MHz nominal frequency transducers. The two-dimensional transthoracic echocardiography and M-mode was obtained at a scanning speed of 200 mm adjusted according to heart rate. Tricuspid annular plane systolic excursion (TAPSE) was evaluated and used as an indicator of RV function.

### Sample collection

The animals from two experimental groups (Control and PAH) were euthanized by decapitation on the 25th day after the injection of MCT. After euthanasia, the heart and RV were dissected and processed for the analyzes of interest, as described below.

### Analysis of chemical elements

A scanning electron microscope (Leo 1430 V P, Carl Zeiss, Jena, Thuringia, Germany) with an attached x-ray detector system (Tracor TN5502, Middleton, WI, USA) was used, as previously described (Bastos et al. 2020), to obtain images of the distribution of chemical elements and to determine the proportion of carbon (C), sodium (Na), potassium (k), calcium (Ca), iron (Fe), magnesium (Mg), copper (Cu), zinc (Zn), and selenium (Se) in cardiac tissue (RV) in a model of MCT- induced PAH. For this end, fragments (4X3X2.5 mm) from the RV were fixed in 2.5% glutaraldehyde, dehydrated in ethanol, submitted to critical point drying (CPD 030, Bal-tec, Witten, North Rhine-Westphalia, Germany) and coated with evaporated carbon (Quorum Q150 T, East Grinstead, West Sussex, England, UK). The EDS microanalysis was performed at × 1000 magnification with an accelerating voltage of 20 kV.

### Protein expression

The expression of type 2 ryanodine receptors (RyR2), Phospholamban (PLB), Phospho-Phospholamban, sarcoplasmic/endoplasmic reticulum Ca^2+^-ATPase (SERCA2) and sodium/calcium exchanger (NCX1) proteins was determined by Western blotting, as previously described (Pelozin et al. 2022). Frozen fragments of the RV (100 mg) were homogenized in RIPA buffer, composed of 150 mM NaCl, 50 mM Tris–HCl (pH 8.0), 0.1% sodium dodecyl sulfate, 1% NP-40, in addition to a protease and phosphatase inhibitor cocktail (1:100; Sigma-Aldrich, St. Louis, MO; PhosSTOP 1:10; Roche, Basel, Switzerland). Insoluble cardiac tissues were removed by centrifugation at 3,000 g, 4 °C, for 10 min. Samples were loaded and subjected to SDS-PAGE on 8–10% polyacrylamide gels. After electrophoresis, proteins were electrotransferred to a nitrocellulose membrane (Amersham Biosciences, Piscataway, NJ). Equal loading of samples (40 μg) and even transfer efficiency were monitored with the use of 0.5% Ponceau S staining of the blot membrane. The blot membrane was then incubated in a blocking buffer (5% nonfat dry milk, 10 mM Tris·HCl, pH 7.6, 150 mM NaCl, and 0.1% Tween 20) for 2 h at room temperature and incubated with specific antibodies overnight at 4 °C. The Odyssey^®^ Fc Imaging System (LI-COR; Lincoln, NE, USA) was used to detect labeled proteins.

The following primary antibodies were used: α- Tubulin (#2144) and GAPDH (#97,166), both from Cell Signalling Technology (Danvers, MA, USA). RyR2 ((#TA5-87,416), PLB (#PA5-85,268), p-PLB (Ser16, Thr17) (#711,401), SERCA2 (#MA3-919) were from Invitrogen Life Technologies (Strathclyde, UK). NCX1 (#151,608) were from Abcam (Cambridge, UK). For all primary antibodies were used at 1:1000 dilution, the secondary antibodies used were IRDye^®^ 680RD or 800CW (1:15,000 LICOR, Lincoln, NE, USA). Bands were analyzed with Image J software (ImageJ based on NIH Image).

### Isolation of right ventricular myocytes

Myocytes from the RV were enzymatically isolated as previously described (Natali et al. 2001). Briefly, after euthanasia, the heart was rapidly dissected and attached to a Langendorff-retrograde perfusion system via aorta and perfused with Tyrode solution containing (in mM): 130 NaCl, 1.43 MgCl_2_, 5.4 KCl, 0.75 CaCl_2_, 5.0 Hepes, 10.0 glucose, 20.0 taurine and 10.0 creatine, pH 7.4 until for about 5 min. The Tyrode solution was thus exchanged to Tyrode solution containing EGTA (0.1 mM) for 5 min. Subsequently, the heart was perfused with Tyrode solution containing 1 mg/ml collagenase type II (Worthington, USA) and 0.1 mg/ml protease (Sigma-Aldrich, USA) for about 12 min. Following this process, the RV of the digested heart was separated and cut into small fragments. These fragments were placed into a conical flask containing the enzymatic solution (collagenase and protease) and mechanically separated by shaking the flask for 5 min. Thereafter, the dispersed cells were separated from the non-dispersed tissue by filtration. After centrifugation, the resulting cells were suspended in Tyrode solution. The non-dispersed tissue was subjected to the mechanical dispersion process again. All solutions used in the isolation procedure were oxygenated (100% O2—White Martins, Brazil) and maintained at 37 °C. The isolated cells were stored at 5 °C until use and were utilized within 2–3 h after isolation.

### Measurement of cardiomyocyte contractility

The contractile function of RV myocytes was measured by using an edge detection system (Ionoptix, Milton, USA) mounted on an inverted microscope (Nikon Eclipse—TS100, Japan) as previously described [14]. In summary, myocytes were placed in a bath on the stage of the inverted microscope and superfused with Tyrode solution containing (in mM): 137 NaCl, 5.4 KCl, 0.33 NaH_2_PO_4_, 0.5 MgCl_2_, 5 HEPES, 5.6 glucose, and 1.8 CaCl_2_, (pH 7.4) at 37 ± 1 °C. Only cardiomyocytes that presented a sarcomere with a clear and regular striated pattern, that did not contract spontaneously in the absence of external stimulation and that responded to 1 Hz stimulation with a single contraction were analyzed. The RV myocytes were externally stimulated (40 V, 5 ms duration) to contract at a frequency of 5 Hz using an electric field stimulator (Myopacer, Ionoptix, Milton, USA). The cardiomyocytes were then visualized on a computer monitor using a CCD camera (Myocam, Ionoptix, Milton, USA) and the images of cell contractions were recorded. From the recordings, the amplitude of cell shortening was evaluated as a percentage of the resting cell length. Cell departure velocity and return velocity were measured as the maximal rate of sarcomere shortening and relaxation, respectively as a percentage of the control group (Hobai et al. 2016). All these parameters were analyzed using the IonWizard 6.3 software (Ionoptix, Milton, USA).

### Statistical analysis

The normality of the data was tested using the Shapiro–Wilk test. Differences between groups were tested using Student’s t test. Data are presented as mean ± SD. P < 0.05 was considered for statistically significant differences. All analyzes were performed using GraphPad Prism, version 6.01 (San Diego, CA, USA).

## Results

There was no difference between the groups in initial body weight; however, the PAH animals showed a reduction in final weight (25rd day after MCT) compared to the control group (Table 1). Additionally, the PAH group animals exhibited an increase in heart weight, RV, and their respective ratios compared to the control group. Regarding the resistance exercise tolerance test, there was no difference before the MCT application. However, the PAH group animals showed a reduction in maximum carrying load compared to the control group.

**Table 1:** Body weight, organ weight, and maximum carrying load in the experimental groups

	Control	PAH	p-value
Initial BW (g)	201.4 ± 05.77	203.3 ± 09.14	0.6576
Final BW (g)	294.9 ± 21.06	225.4 ± 14.80	< 0.0001
HW (g)	1.012 ± 0.059	1.373 ± 0.091	< 0.0001
RVW (g)	0.202 ± 0.020	0.419 ± 0.042	< 0.0001
HW/TL (mg/mm)	0.005 ± 0.000	0.011 ± 0.001	< 0.0001
RVW/TL (mg/mm)	0.005 ± 0.001	0.012 ± 0.001	< 0.0001
Initial MCL (g/g)	1.09 ± 0.114	1.07 ± 0.053	0.6608
Final MCL (g/g)	1.23 ± 0.154	0.974 ± 0.081	0.0019

Data are means ± SD of 7 rats in each group

*BW* body weight, *HW* heart weight, *MCL* maximum carrying load, *RVW* right ventricle weight, *TL* tibia length

**Fig. 1:**
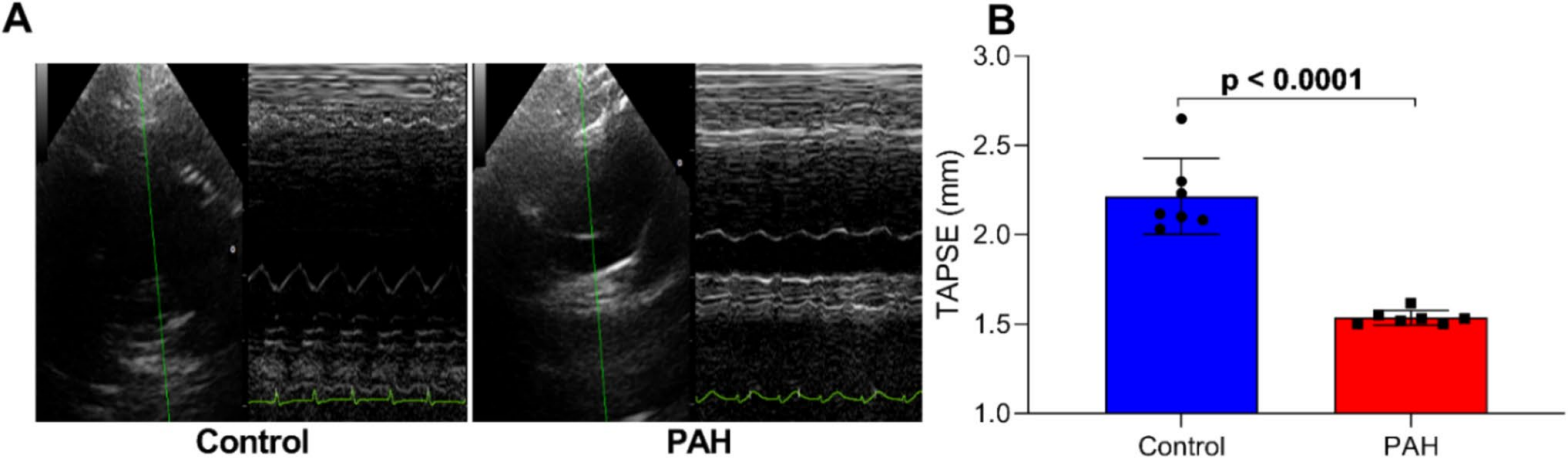
Effects of PAH on cardiac function. **A** Representative images of tricuspid annular plane systolic excursion (TAPSE). **B** TAPSE measured in 24 days after injection of MCT. Data are mean ± SD of 7 rats in each group. PAH, pulmonary arterial hypertensions. Student t-test

The systolic function of the RV was assessed using TAPSE (Fig. 1). In panel A, representative images show that animals in the control group exhibited a wide and rhythmic excursion of the tricuspid annulus during systole, indicating normal RV function. In contrast, animals with PAH showed visibly reduced excursion of the tricuspid annulus, with smaller and irregular oscillations, suggesting impaired RV function. These findings are confirmed in panel B, where animals with PAH demonstrate a significant reduction in TAPSE compared to the control group animals.

**Fig. 2:**
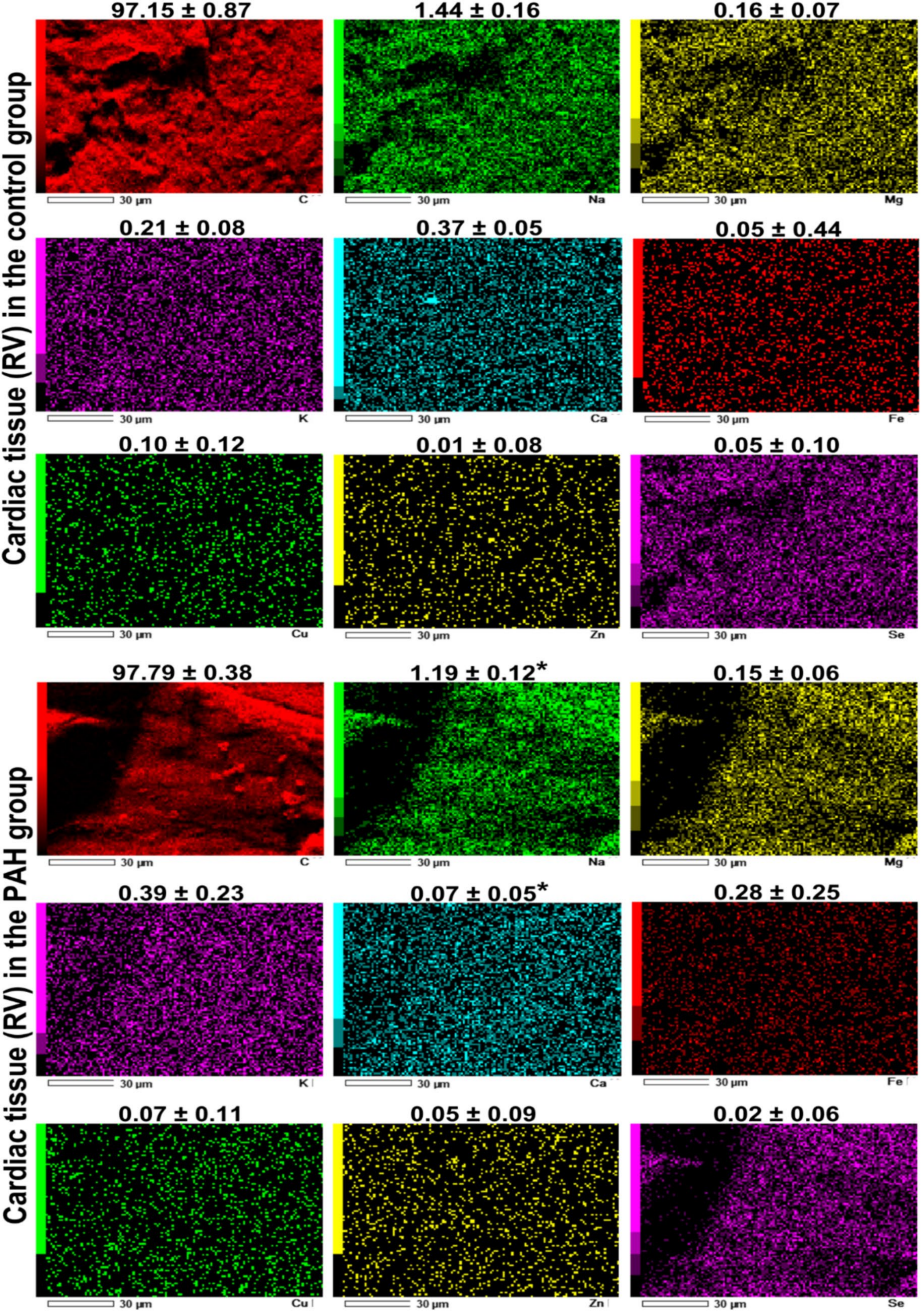
Elemental map of the right ventricle (RV) from control (upper panel) and PAH (lower panel) animals (1000 × magnification). The graphs represent the X-ray emission spectrum for the analyzed elements, and the values above the images indicate the elemental concentration in weight percent. Data are means ± SD of 6 animals in each group. C, carbon; Na, sodium; K, potassium; Ca, calcium; Mg, magnesium; Cu, copper; Zn, zinc; Se, selenium. Student t-test. *P < 0.05 vs. control group

Having confirmed the impairment of RV function by echocardiographic examination in this model of monocrotaline-induced PAH, we mapped the tissue electrolytes in the RV cardiac tissue by Energy dispersive X-ray spectroscopy associated with scanning electron microscopes and evaluated the effect of PAH on these elements (Fig. 2). The mapping showed that although the mineral distribution was homogeneous, animals with PAH have lower Ca and Na density compared to control animals.

**Fig. 3:**
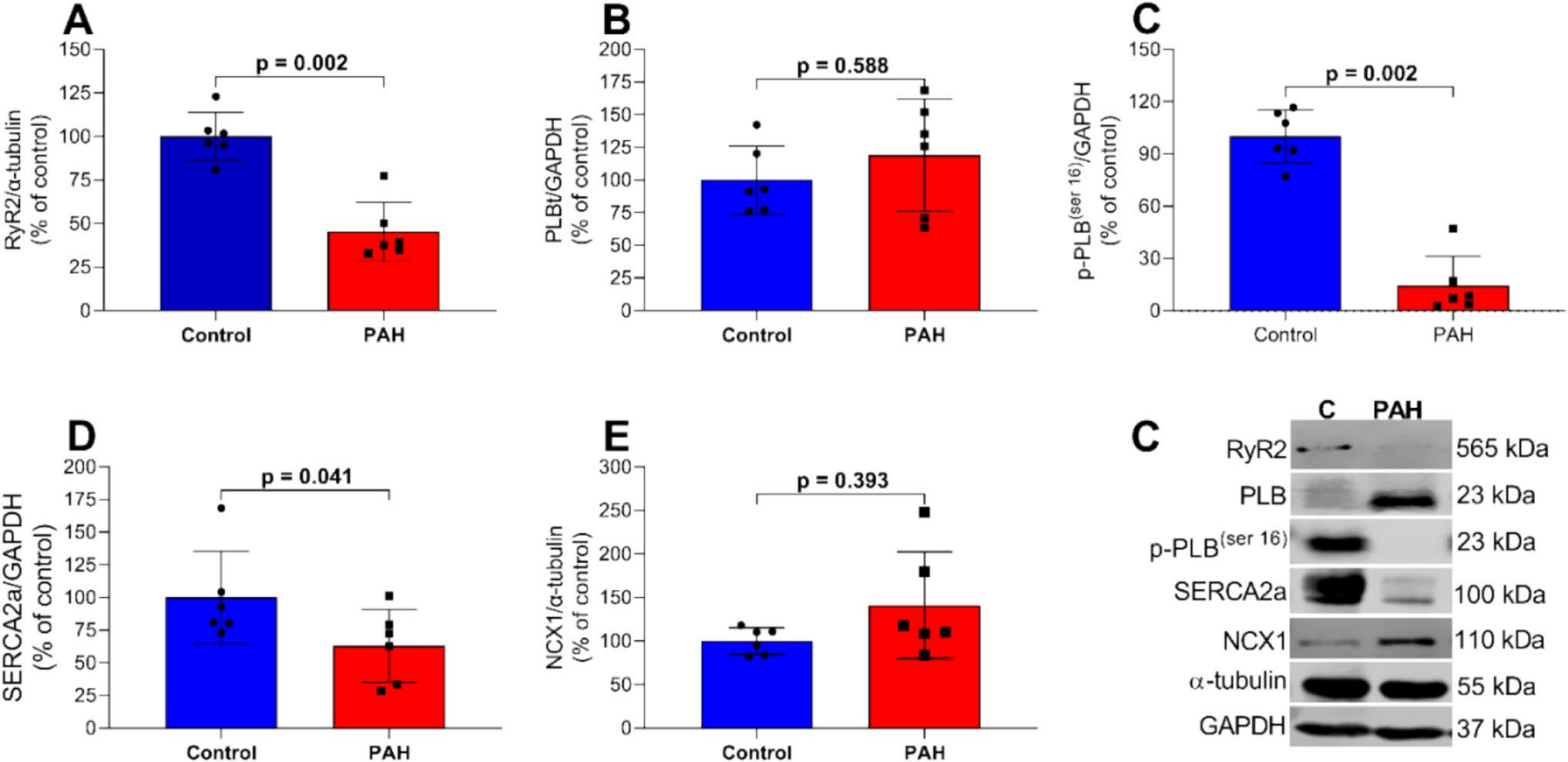
Effect of experimental PAH on the expression of Ca^2+^ transient regulatory proteins. **A** Total phospholamban (PLBt). **B** Phospholamban phosphorylated to serine 16 (PLBser16). **C** Sarcoplasmic/endoplasmic reticulum Ca^2+^-ATPase (SERCA2). (D) sodium/calcium exchanger (NCX1). Data are means ± SD of 8 animals in each group. Student t-test

Figure 3 shows the results regarding the effect of PAH on the proteins regulating Ca^2+^ transient and consequently excitation–contraction coupling. The animals in the PAH group showed reduced expression of the proteins RyR2, p-PLBser16 and SERCA2a compared to the animals in the control group.

**Fig. 4:**
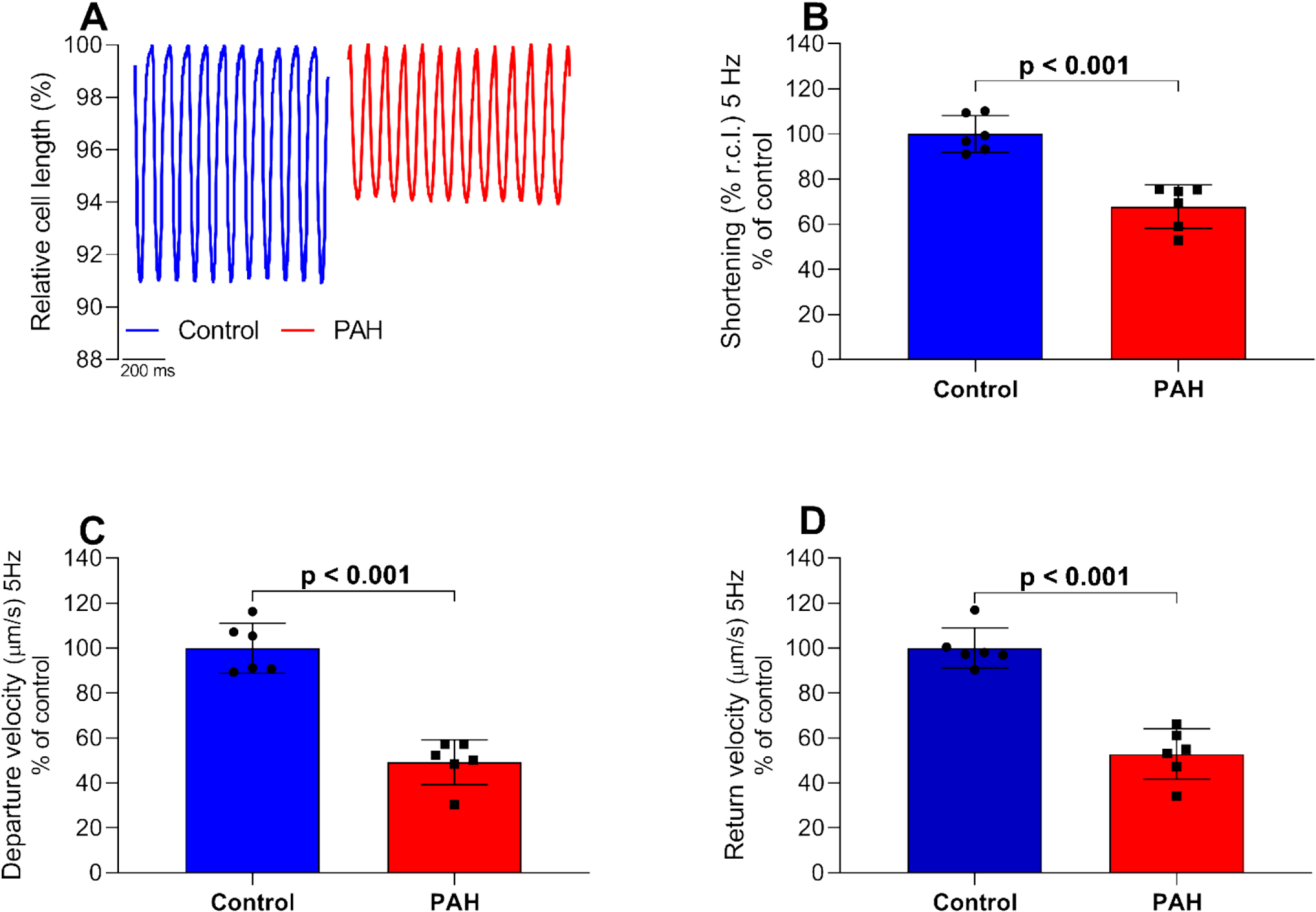
Effects of PAH on right ventricular myocyte contractility. **A** Typical cell shortening traces (stimulation 5 Hz). **B** Amplitude of shortening. **C** Departure velocity. **D** Return velocity. Values are means ± SD of 6–10 cells per animal in each group (n = 6 rats in each group). PAH, pulmonary arterial hypertensions. Student t-test

Figure 4 presents the results of the effect of PAH on the contractility of isolated cardiomyocytes stimulated at 5 Hz. In panel A, the traces show that animals in the control group exhibited a wide and rhythmic amplitude of movement, indicating normal cellular function. In contrast, animals with PAH exhibited a smaller and irregular amplitude of movement, suggesting impaired cellular function. These findings are confirmed in panels B, C, and D, where animals in the PAH group showed a decrease in contraction amplitude, contraction velocity, and relaxation velocity compared to those in the control group. These findings highlight the adverse impacts of pulmonary arterial hypertension on cellular contractility.

## Discussion

The MCT-induced PAH animal model is widely adopted by researchers worldwide due to its technical simplicity, ease of experiment replication, and relatively low cost compared to other models (Gomez-Arroyo et al. 2012; Gewehr et al. 2020; Kim and Yoo 2016). This model is capable of mimicking several features of human PAH, including pathological remodeling, smooth muscle cell proliferation, endothelial dysfunction, increased expression of inflammatory cytokines, and primarily RV dysfunction (Thenappan et al. 2018; Hill et al. 2017). In this study, we confirmed that this PAH model exhibits RV hypertrophy and severe systolic dysfunction, one of the most severe characteristics of human PAH. Additionally, associated with the functional and structural alterations of the RV, PAH animals showed reduced physical exercise tolerance. Beyond confirming the classical phenotype of the model, the primary novelty of the present study lies in utilizing this well-characterized model to uncover tissue-level ionic imbalances using EDS-SEM. This approach allows us to propose a direct link between the depletion of ionic reserves in the cardiac matrix and the contractile failure observed in the disease.

Once the cardiac dysfunction was characterized, we mapped the elemental distribution in the RV tissue using EDS-SEM. This technique revealed a significant relative reduction in Ca and Na density in the PAH myocardium. In this context, ‘relative reduction’ indicates a decrease in the abundance of these ions within the analyzed tissue matrix relative to the total elemental spectrum. This suggests a depletion of the tissue’s ionic reserves, which are fundamental for maintaining the electrochemical gradients required for excitation–contraction coupling (Bers 2002; Despa and Bers 2013).

The finding of reduced tissue Na levels is particularly intriguing when contrasted with classical heart failure literature. While studies focusing solely on the intracellular compartment often report elevated cytosolic Na⁺ due to leakage or Na⁺/K⁺ pump failure (Despa and Bers 2013; Schwinger et al. 2003; Aronsen et al. 2013; MacLeod 2023), our EDS data reflects the in situ tissue composition. We propose that the overall depletion of Na observed here may compromise the transmembrane concentration gradient necessary for the rapid Na⁺ influx during depolarization. This aligns with pivotal concepts demonstrating that strict Na⁺ and K⁺ homeostasis, as well as proper ion pump function, are essential for sustaining contractility and preventing fatigue in the mammalian myocardium (Swift et al. 2007; Sejersted and Sjøgaard 2000). Consequently, even if intracellular handling is impaired, a lack of available Na in the tissue microenvironment could further blunt the action potential upstroke and conduction velocity, contributing to the systolic failure observed.

Similarly, the reduction in tissue Ca aligns with the impairment of the intracellular Ca^2^⁺ handling machinery. In our study, the depletion of Ca reserves was accompanied by the downregulation of critical regulatory proteins, including RyR2, SERCA2a, and p-PLB. This creates a ‘double hit’ scenario: not only is the expression of proteins responsible for Ca^2^⁺ cycling reduced (i.e., slower reuptake and weaker release) but the availability of Ca itself in the tissue is compromised. Although NCX1 expression remained unchanged, the reduced substrate availability (Na and Ca) likely renders this compensatory mechanism insufficient to preserve contractility (Bers 2002; Pott et al. 2011).

Ultimately, these ionic and molecular alterations culminated in the functional collapse of the cardiomyocytes. Our data from isolated myocytes showed significant impairments in amplitude, as well as contraction and relaxation velocities (at 5 Hz stimulation). This confirms that the mechanical inefficiency of the RV in this model is not merely a structural consequence of fibrosis or hypertrophy (Soares et al. 2019, 2023, 2025), but is mechanistically underpinned by the disruption of the ionic landscape and excitation–contraction coupling proteins (Soares et al. 2025, 2023). Thus, EDS-SEM proves to be a valuable tool for identifying these broad ionic deficits that precipitate the cellular dysfunction driving heart failure in PAH. Crucially, the translational potential of this technology extends to human pathophysiology. Given the well-established link between ionic shifts and functional impairment (Bers 2002; Despa and Bers 2013; Aronsen et al. 2013; Swift et al. 2007; Sejersted and Sjøgaard 2000), applying EDS-SEM to human endomyocardial biopsies could allow for the assessment of ‘ionic reserves’, offering a novel marker for myocardial remodeling that complements traditional histological analysis.

## Conclusion

The results of this study demonstrate that MCT-induced PAH is associated with ionic imbalances in the RV, characterized by reduced Ca and Na levels. These ionic alterations were accompanied by changes in the expression of proteins involved in the regulation of excitation–contraction coupling. Consequently, a significant impairment in isolated RV cardiomyocyte contractility and cardiac function was observed. Taken together, these findings suggest that ionic imbalances play a central role in the contractile dysfunction associated with PAH, contributing to the progression of cardiac remodeling observed in this experimental model.

## Data Availability

Data will be made available on request.
